# Sepsis due to *Streptococcus pneumoniae* associated with secondary hemophagocytic lymphohistiocytosis in a splenectomized patient for spherocytosis: A case report

**DOI:** 10.1097/MD.0000000000007520

**Published:** 2017-07-14

**Authors:** Victoria Birlutiu, Rares Mircea Birlutiu

**Affiliations:** a Faculty of Medicine Sibiu, Lucian Blaga University of Sibiu; b Academic Emergency Hospital Sibiu, Infectious Diseases Clinic, Sibiu; c Spitalul Clinic de Ortopedie-Traumatologie si TBC Osteoarticular “Foisor” Bucuresti, Romania.

**Keywords:** hemophagocytic lymphohistiocytosis, HLH, splenectomized patient, *Streptococcus pneumoniae*

## Abstract

**Rationale:**

Hemophagocytic lymphohistiocytosis (HLH) is a syndrome that is characterized by an inappropriate hyperinflammatory immune response – primary, as a consequence of a genetic defect of NK cells and cytotoxic T lymphocytes or – secondary, in the progression of infections, rheumatic or autoimmune diseases, malignancies or metabolic diseases.

**Patient concerns:**

We present the case of a secondary HLH due to Streptococcus pneumoniae infection in a splenectomised patient for spherocytosis, a 37-year-old patient who was splenectomised in childhood for spherocytosis, without immuneprophylaxis induced by antipneumococcal vaccine.

**Outcomes:**

He developed a severe pneumococcal sepsis associated with secondary HLH, with unfavorable outcome and death.

**Lessons:**

To our knowledge, just 2 similar cases had been published in the literature, none in which the secondary HLH was the consequence of an invasive pneumococcal infection in a splenectomized patient for spherocytosis, and the association of splenectomy with HLH is surprizin.

## 1 Introduction

Hemophagocytic lymphohistiocytosis (HLH) is a syndrome that is characterized by an inappropriate hyper-inflammatory immune response – primary, as a consequence of a genetic defect of NK cells and cytotoxic T lymphocytes or – secondary, in the progression of infections, rheumatic or autoimmune diseases, malignancies or metabolic diseases. Among HLH-related infections, the most common are the viral infections: Epstein–Barr virus, cytomegalovirus, other herpes viruses, the viruses of hepatitis B and C, etc., followed by bacterial, parasitic, or fungal infections. Association of secondary HLH to *Streptococcus pneumoniae* infection in a patient splenectomized for spherocytosis has not been described so far.

## 2 Case report

**Figure 1:**
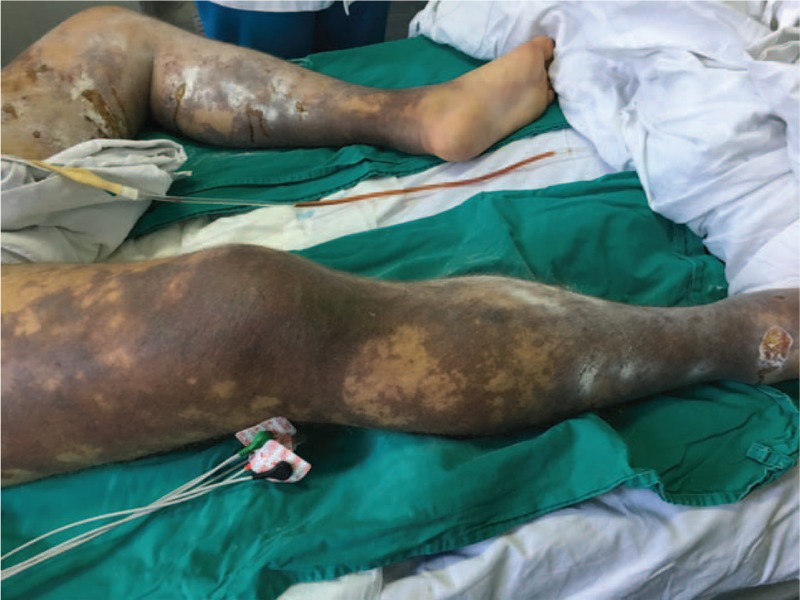
Picture from the emergency room that presents the purpura on the legs and skin lesions.

We present the case of a male Caucasian patient, aged 37 years, splenectomized for spherocytosis since the age of 4, with no prophylaxis of meningococcal, and pneumococcal infections through vaccination, that was brought to the emergency room for fever, diarrhea, vomiting, rash skin, myalgia, anuria, and marked alteration of his general condition. At the time of admission, on physical examination, the following changes were noticed: facial erythema, purpura on the legs (see Fig. 1), cyanosis of the extremities, jaundice, left basilar crackles, heart rate of 130 beats per minute, blood pressure of 140/100 mm Hg, hepatomegaly, and anuria. The laboratory investigations that were performed revealed the following alterations (at admission and in evolution) that are presented in Table 1.

**Table 1:**
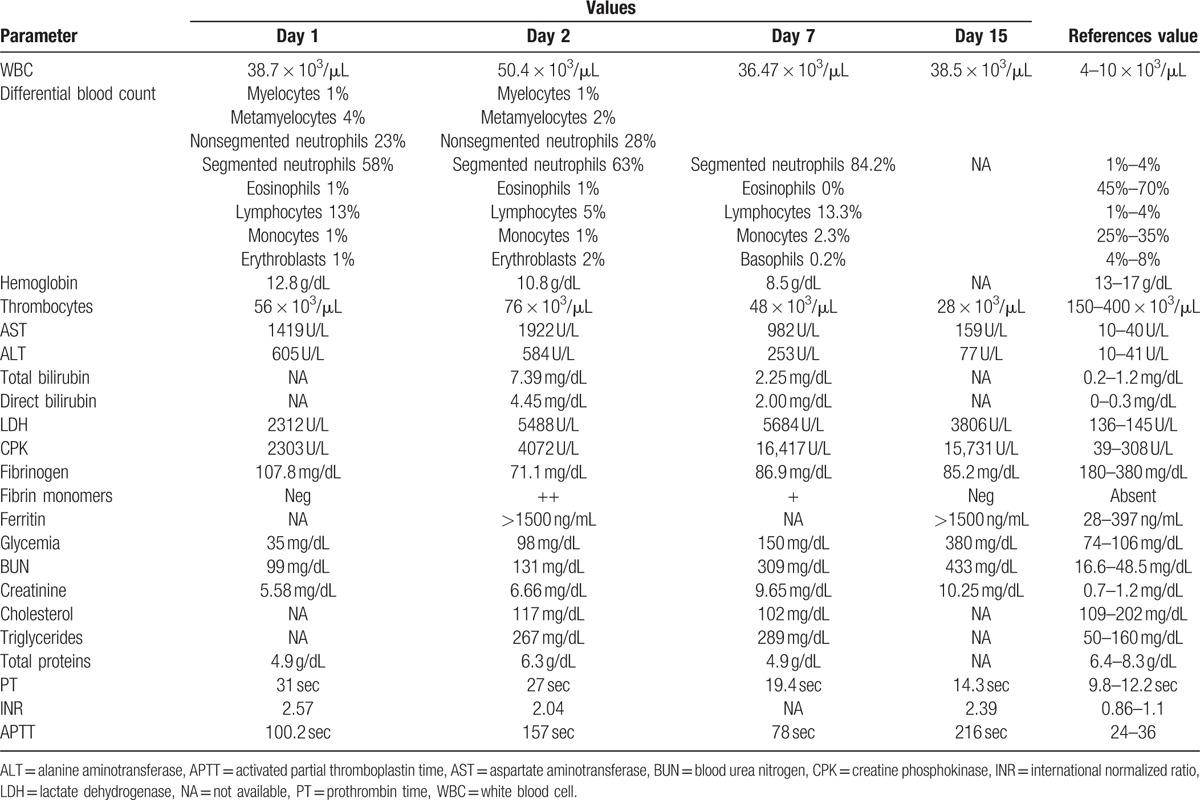
Laboratory studies.

Peripheral smear revealed normochromic normocytic red blood cells, as well as microspherocytes and spherocytes, frequent polychromatophilic macrocytes; erythrocytes with Howell–Jolly bodies (splenectomy), rare dacryocytes (teardrop cells), and schistocytes. Polymorphonuclears with vacuolated cytoplasm are present (toxic appearance), diplo-, encapsulated, and intra- and extracellular gram-positive cocci. Bone marrow sample was harvest and on hematoxylin-eosin stain an increased number of activated macrophages with prominent hemophagocytosis of hematopoietic elements was revealed. Blood cultures and urine cultures were positive for *S pneumoniae*, resistant to benzylpenicillin, chloramphenicol, erythromycin, trimethoprim/sulfamethoxazole, tetracycline, cefotaxime, and intermediate resistance to ceftriaxone, imipenem, sensitive to ofloxacin, vancomycin, moxifloxacin, quinupristin/dalfopristin, levofloxacin, linezolid, rifampicin, sparfloxacin, pristinamycin, amoxicillin, and telithromycin. Viral, parasitic etiologies were excluded, as well as rheumatic diseases, malignant tumors, which may be involved in secondary HLH.

A cardiac ultrasound was performed and revealed no suggestive images of infectious endocarditis or valvular heart disease. Initially, the chest radiography revealed no changes, but in evolution, it showed bilateral alveolar condensation and left pleural effusion.

The case was interpreted as sepsis due to a multidrug-resistant *S pneumoniae* associated with consumption coagulopathy (bleeding at venepuncture site and epistaxis), acute liver failure, acute renal failure by myoglobinuria, and HLH. In evolution, acute respiratory failure occurred, for which endotracheal intubation of the patient was performed.

**Figure 2:**
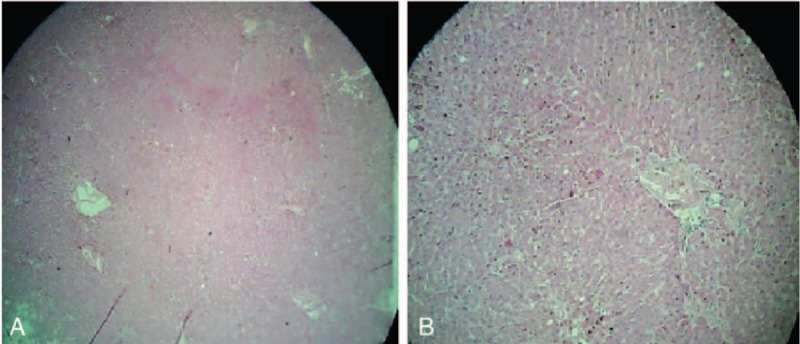
Hematoxylin and eosin-stained sections of liver. Magnifications: ×40 (A) and ×100 (B).

**Figure 3:**
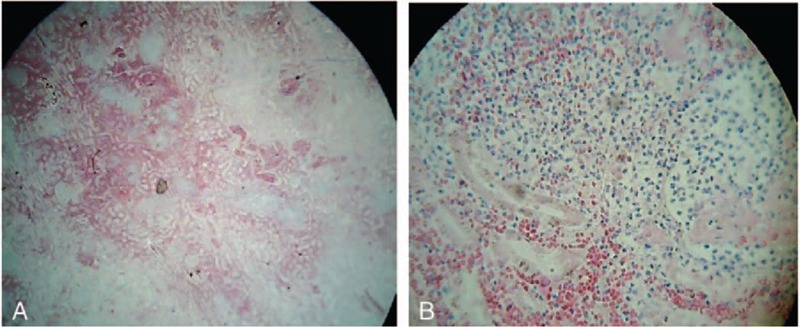
Hematoxylin and eosin-stained sections of kidney. Magnifications: ×40 (A) and ×100 (B).

**Figure 4:**
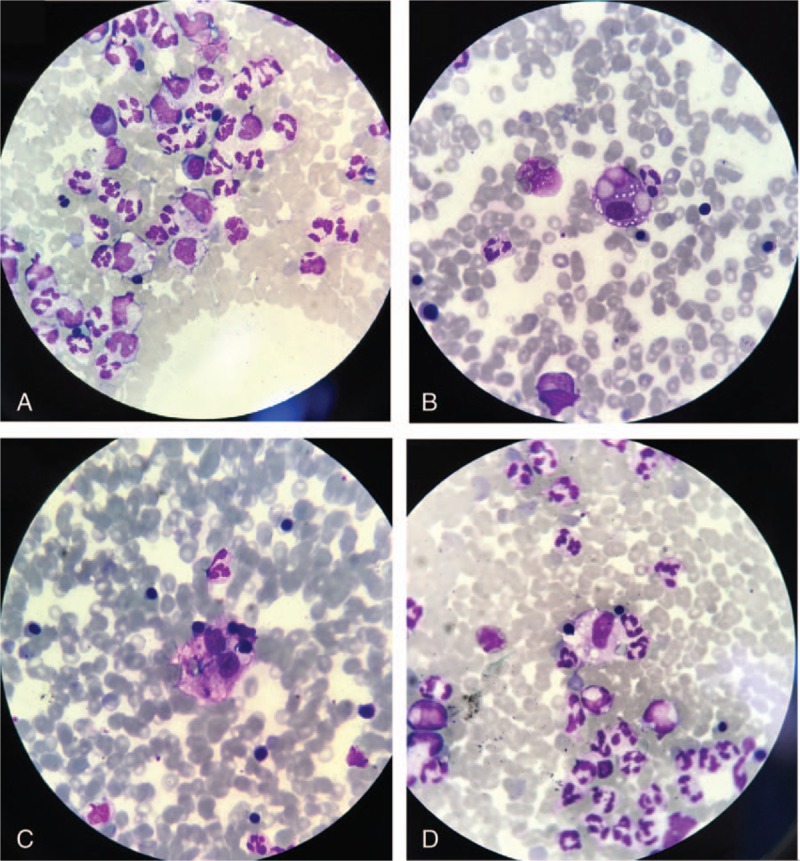
May-Grünwald Giemsa-stained sections of bone marrow. Magnifications: ×100 (A–D).

The treatment was started with infusions of macromolecular solutions, hydro-electrolytic rebalancing, packed red blood cells, fresh frozen plasma, and antibiotics – initially, with ultrabroad-spectrum antibiotics, meropenem 2 g/day associated with linezolid 2 × 600 mg/day, thereafter treatment continued with linezolid associated with moxifloxacin 400 mg/day according to antibiogram results, dexamethasone, etoposides 150 mg/m^2^/day, 3 days, anidulafungin, intravenous immunoglobulin, and daily hemodialysis sessions throughout hospitalization. The evolution was unfavorable with coma, Glasgow Coma Scale of 3, and quadriplegia, the occurrence of bronchopneumonia required endotracheal intubation. During hospitalization, the patient was anuric. Patient death occurred on day 15 of hospitalization. Hematoxylin-eosin and immunohistochemically stainings of liver biopsies taken during the anatomopathological examination revealed the following changes: massive infiltration of portal tract and sinusoids by mononuclear cells. The CD68 stain shows numerous large, irregularly shaped CD68+ cells as being macrophages, cells that are localized both in the portal tract and sinusoids, and with an increased phagocytic activity on lymphocytes, erythrocytes, and polynuclear cells. On CD8 stain, numerous CD8+ lymphocytes were revealed. The conclusion was: the described aspect is in concordance with the diagnosis of hemophagocytic lymphohistiocytosi. Autopsy examination also revealed bilateral renal papillae necrosis secondary to myoglobinuria, and the presence of hemophagocytosis in bone marrow, and lymph nodes Figs. 2–4.

## 3 Discussions

This case brings into question the risk of splenectomised patients to develop severe systemic infections with encapsulated bacteria, against which the vaccine prophylaxis is essential – for *S pneumoniae*, *H influenzae*, and *Meningococcus*. Lack of vaccination in this patient has enabled the development of severe infections with multidrug-resistant *S pneumoniae* and the induction of secondary HLH, characterized by an uncontrolled inflammatory response, resulting in patient's death.

Primary HLH has a family, autosomal recessive transmission, and is present in 50,000 new-borns annually. Secondary HLH may be induced by viral (29%), bacterial, or parasitic infections (20%), autoimmune or rheumatic diseases (7%), cancers (27%), and metabolic diseases or immunodeficiency syndromes (6%).^[1]^ Among viral causes, the most common is associated with Epstein–Barr virus. There are also described associations with cytomegalovirus, herpes simplex,^[2,3]^ varicella-zoster virus,^[4]^ herpes virus 8, and associated with HIV infection.^[5]^ There were also published associations with hepatitis B and C viruses,^[6,7]^ influenza viruses,^[8]^ enteroviruses,^[9]^ rotavirus, severe acute respiratory syndrome virus,^[10]^ hemorrhagic fevers,^[11–15]^ HIV,^[15–18]^ and so on. Among bacterial infections associated with HLH, there were published associations with *Borrelia*,^[19]^*Babesia* sp,^[20]^*Bartonella* sp,^[21]^*Brucella* sp,^[22]^ Q fever,^[23]^*Leptospira* sp,^[24]^*Listeria monocytogenes*,^[25]^*Mycoplasma pneumoniae*,^[26]^ and mycobacteria.^[27–32]^ The most frequently described parasitic causes responsible for secondary HLH are: *Leishmania* sp,^[33,34]^ malaria,^[35–37]^ and *Toxoplasma gondii*.^[38,39]^ Fungal infections are found to be associated with secondary HLH in HIV-infected patients such as *Cryptococcus neoformans*,^[40]^*Candida* spp,^[41]^ or in patients with renal transplantation-association with disseminated histoplasmosis.^[42]^

Of the defining HLH criteria established by the Histiocyte Society, respectively, fever, splenomegaly, cytopenia (on at least 2 lines in peripheral blood), hypofibrinemia, hyperferritinemia, hypertriglyceridemia, presence of hemophagocytosis in bone marrow or lymph node, reduction/absence of NK cells activity, and increase in the concentration of soluble IL-2 receptor, CD25,^[43]^ the patient had 5 defining criteria for HLH. The presence of consumption coagulopathy evidenced by the presence of fibrin degradation products is associated with hypofibrinemia and thrombocytopenia in HLH, as well as the liver damage-elevated transaminases, hyperbilirubinemia, activated partial thromboplastin time prolongation. These changes are also present within the septic context, their strict delimitation is not possible.

Although the diagnosis of HLH was early, the administration of dexamethasone, intravenous immunotherapy, and administration of etoposides have not improved prognosis, the patient's death occurring on the 15th day of hospitalization. Renal insufficiency due to bilateral renal papillary necrosis has been associated with myoglobinuria as a result of septic myositis, demonstrated by high levels of creatine phosphokinase. There have been described situations in which *S pneumoniae* infections are responsible for the death of patients with primary HLH.^[44]^ Two cases of HLH associated with a pneumococcal infection had been published (see Table 2). HLH association with hereditary spherocytosis is found in association with viral infections, such as parvovirus B19^[47]^ or Epstein–Barr virus. Moreover, splenectomy is described as a therapeutic method in refractory HLH cases.^[48–50]^

**Table 2:**
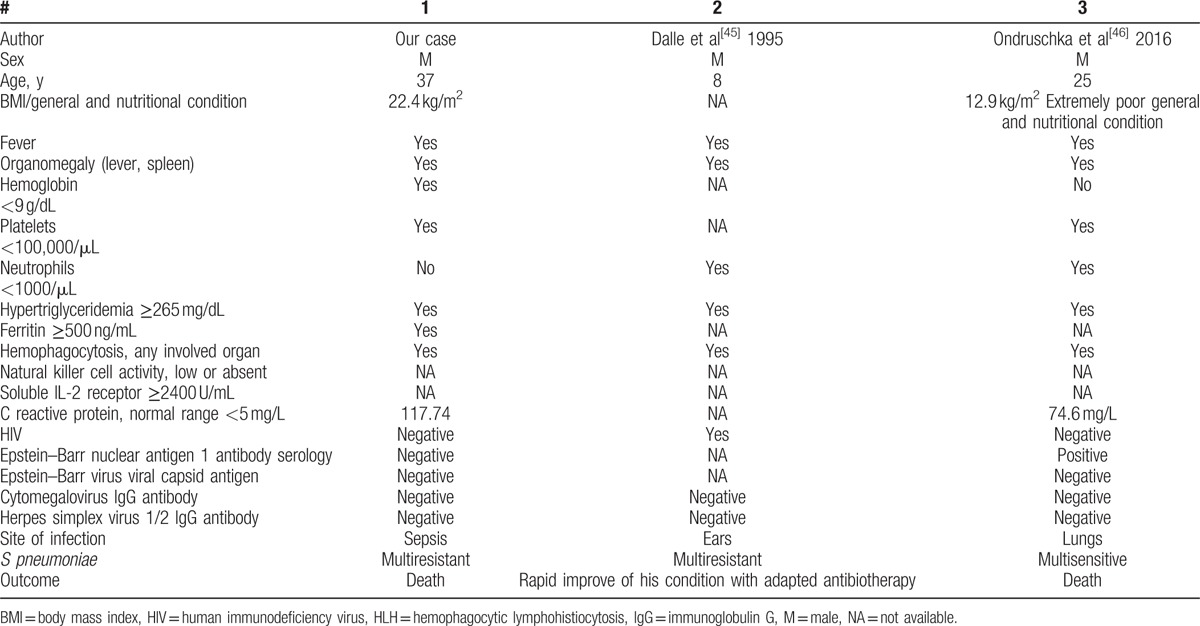
Clinical and biological characteristics of the 3 cases with HLH due to *S pneumoniae.*

### 3.1 Informed consent

Written informed consent was obtained from the patient's next of kin (from his father) for publication of this case report and any accompanying images. The study was accepted by the Ethics Committee of the hospital and they encouraged publishing the article. A copy of the written consent is available for review by the Editor-in-Chief of this journal.

## 4 Conclusion

The association of sepsis due to *S pneumoniae* with secondary HLH in a splenectomized patient for spherocytosis should be taken into consideration in similar cases like ours. To our knowledge, no similar cases had been published in the literature, in which the secondary HLH was the consequence of an invasive pneumococcal infection in a splenectomized patient for spherocytosis, and the association of splenectomy with HLH is surprizing. Other similar observations are necessary in the future.
